# Double Bond Stereochemistry Influences the Susceptibility of Short-Chain Isoprenoids and Polyprenols to Decomposition by Thermo-Oxidation

**DOI:** 10.1007/s11745-015-3998-8

**Published:** 2015-03-05

**Authors:** Ewa Molińska, Urszula Klimczak, Joanna Komaszyło, Dorota Derewiaka, Mieczysław Obiedziński, Magdalena Kania, Witold Danikiewicz, Ewa Swiezewska

**Affiliations:** 1 Department of Lipid Biochemistry, Institute of Biochemistry and Biophysics, Polish Academy of Sciences, Pawinskiego 5a, 02-106 Warsaw, Poland; 2 Faculty of Food Sciences, Warsaw University of Life Sciences, Nowoursynowska 159, 02-776 Warsaw, Poland; 3 Laboratory of Mass Spectrometry, Institute of Organic Chemistry, Polish Academy of Sciences, Kasprzaka 44/52, 01-224 Warsaw, Poland

**Keywords:** Isoprenoid, Oligoprenol, Polyprenol, Thermo-oxidation, Oxidation products

## Abstract

Isoprenoid alcohols are common constituents of living cells. They are usually assigned a role in the adaptation of the cell to environmental stimuli, and this process might give rise to their oxidation by reactive oxygen species. Moreover, cellular isoprenoids may also undergo various chemical modifications resulting from the physico-chemical treatment of the tissues, e.g., heating during food processing. Susceptibility of isoprenoid alcohols to heat treatment has not been studied in detail so far. In this study, isoprenoid alcohols differing in the number of isoprene units and geometry of the double bonds, β-citronellol, geraniol, nerol, farnesol, solanesol and Pren-9, were subjected to thermo-oxidation at 80 °C. Thermo-oxidation resulted in the decomposition of the tested short-chain isoprenoids as well as medium-chain polyprenols with simultaneous formation of oxidized derivatives, such as hydroperoxides, monoepoxides, diepoxides and aldehydes, and possible formation of oligomeric derivatives. Oxidation products were monitored by GC-FID, GC-MS, ESI-MS and spectrophotometric methods. Interestingly, nerol, a short-chain isoprenoid with a double bond in the *cis* (*Z*) configuration, was more oxidatively stable than its *trans* (*E*) isomer, geraniol. However, the opposite effect was observed for medium-chain polyprenols, since Pren-9 (di-*trans*-poly-*cis*-prenol) was more susceptible to thermo-oxidation than its all-*trans* isomer, solanesol. Taken together, these results experimentally confirm that both short- and long-chain polyisoprenoid alcohols are prone to thermo-oxidation.

## Introduction

**Fig. 1:**
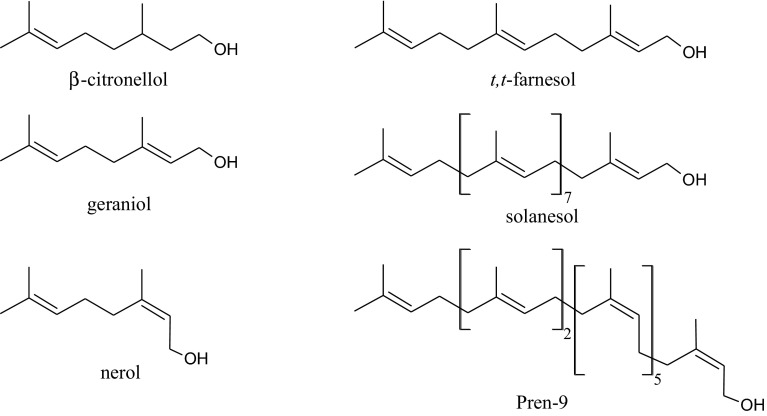
Structures of oligoprenyl and polyprenyl alcohols

Polyisoprenoid alcohols are naturally occurring linear hydrocarbon polymers of five-carbon isoprene units. Polyprenols, present in bacteria and plant photosynthetic tissues, possess an unsaturated bond in the isoprene unit proximal to the hydroxy group (α-end), whereas dolichols, present in mammals, yeast and plant roots, have a saturated one [1]. Besides polyisoprenoids consisting of several up to approximately 100 isoprenoid residues, short-chain oligoprenols (having one to a few isoprene units) (Fig. 1) and high polymers (e.g., gutta-percha and natural rubber with more than 300 isoprene units) can be distinguished [2]. Polyisoprenoids are classified into three main subgroups, di-*trans*-poly-*cis*, tri-*trans*-poly-*cis* and all-*trans* [1], depending on the geometry of the double bond. The most prevalent all-*trans* alcohol is solanesol, i.e., all-*trans* prenol-9 (Fig. 1), accumulated in plants belonging to the *Solanaceae*, e.g., *Nicotiana tabacum* L. [3]. Furthermore, double bonds in natural short-chain oligoprenols might have *trans* (*E*, e.g., geraniol, farnesol and geranylgeraniol) or *cis* (*Z*, e.g., nerol) structures. The hydrophobic character of the polyisoprenoid chain predetermines its localization in cell membranes [1].

Phosphate esters of dolichols and polyprenols fulfill important biological functions, such as cofactors of protein glycosylation in eukaryotic cells or as donors of prenyl residues in the biosynthesis of prenylated proteins and quinones [4]. Recent studies concerning polyisoprenoids are especially interesting, showing that tobacco accumulates polyprenols in response to infections or exogenously applied hydrogen peroxide [5]. An increase in dolichol content has been found in all tissues upon aging; therefore, their involvement in programmed cell death has been postulated. Alternatively, dolichols have been suggested to play an important role in a defense mechanism against adverse environmental conditions [6]. The role of polyisoprenoids in the cell defense strategy could be achieved by either modulation of the physical properties of biological membranes, as shown for model ones [7, 8], or constituting a shield against reactive oxygen species (ROS) [9]. In fact, oxidation products of dolichols were found in living organisms. Ward et al. [10] have identified dolichol species containing an additional one up to even four oxygen atoms in the molecule of dolichol and dolichoic acid in neurologically normal human autopsy brain tissues. Oxidized polyisoprenoids might play a role in cell metabolism, and especially interesting in this context are the results obtained by Bentinger et al. [11, 12], who found that epoxides of solanesol or squalene enhanced CoQ and markedly reduced cholesterol biosynthesis in HepG2 cells.

Studies on the oxidation of short-chain isoprenoids are the way to discover novel compounds, possibly serving as new flavors, fragrances or therapeutically active substances [13]. It should be also kept in mind that oxidation products of some fragrance terpenes have been found to be responsible for contact allergies as they are skin sensitizers [14].

Thermal degradation of terpenes conducted at different temperatures and in the presence or absence of air was studied previously [15, 16]. McGraw and coworkers [15] analyzed the oxidation products of camphene, Δ^3^-carene, limonene and α-terpinene formed during thermal degradation at 120 °C in the presence of air using NMR and/or GC-MS. Their study was conducted to determine which products might be expected under the drying conditions used in wood product processing. As a result, various degradation products resulting from dehydrogenations, epoxidations, double bond cleavages, allylic oxidations, etc., such as epoxides, ketones, aldehydes and alcohols, were found.

The aim of the study was to test the effect of the length of the hydrocarbon skeleton molecule size and geometry of double bonds on the oxidation stability of isoprenoid compounds. In this study, short-chain isoprenoid alcohols (oligoprenols), geraniol, nerol and farnesol, the α-saturated alcohol β-citronellol and the medium-chain polyprenols solanesol and Pren-9 (Fig. 1) were subjected to thermo-oxidation at 80 °C. Thermo-oxidation processes can be considered a simulation of chemical reactions that might occur in living cells, especially upon the increased oxidative potential resulting from adverse environmental conditions.

## Materials and Methods

### Chemicals

Solanesol (all-*trans*-prenol-9) was extracted from tobacco *Nicotiana tabacum* cv. *Samsun* leaves by supercritical fluid extraction (SFE) with carbon dioxide [17]. Pren-9 (di-*trans*-poly-*cis*-prenol-9) was extracted from the wood of *Betula verrucosa*. Polyprenols were purified on silica gel columns with hexane and diethyl ether using a step-wise gradient. Identity of the obtained prenols was verified by the HPLC-UV and ESI-MS (described below) methods by comparison with the solanesol standard purchased from Sigma-Aldrich and Pren-9 standard from the Collection of Polyprenols (Institute of Biochemistry and Biophysics, Polish Academy of Sciences, Warsaw, Poland). Purity of the obtained polyprenols assessed by HPLC-UV was approximately 91 and 98 % for solanesol and Pren-9, respectively. β-Citronellol, nerol, geraniol, *t,t*-farnesol, citral (a mixture of neral and geranial), *tert*-butyl hydroperoxide (*t*BuOOH), pyridine, BSTFA (N,O-bis(trimethylsilyl) trifluoroacetamide)/TMCS (trimethylchlorosilane) (99:1, by vol) and m-chloroperbenzoic acid (mCPBA) were purchased from Sigma-Aldrich. Hexane, methanol, chloroform, isopropanol and ethanol of HPLC grade were purchased from J.T. Baker. All other chemicals of analytical reagent grade were obtained from POCh. Milli-Q water from the Millipore Academic (USA) water purification system was used.

### Heat Treatment

Samples (usually 500 μg) of isoprenoid alcohols, β-citronellol, nerol, geraniol, *t,t*-farnesol, solanesol and Pren-9, in the form of a thin film of pure substance, were subjected to thermo-oxidation at 80 °C for an indicated period of time, from 1 up to 168 h, with free access to the air. After treatment, samples were dissolved in 0.5 ml isopropanol/ethanol (1:1, by vol) and stored at −80 °C until analysis. Experiments were done in triplicate.

### Analysis of Hydroperoxides

Hydroperoxides were measured using the colorimetric ferrous thiocyanate method. Ferrous chloride reagent solution was prepared as described in [18] by mixing an aqueous solution of BaCl_2_·2H_2_O (40 mg/5 ml) and FeSO_4_·7H_2_O (50 mg/5 ml) with 200 μl hydrochloric acid (35–38 %). After preparation, the ferrous chloride reagent was stored for no longer than 5 days at 4–8 °C. The sample (25 µl, diluted if needed) was filled up to 80 µl with isopropanol/ethanol (1:1, by vol), then mixed with ferrous chloride solution prepared as described above (10 µl) and saturated aqueous ammonium thiocyanate (10 µl) and vortexed. After 5 min absorbance at *λ* = 490 nm (Eppendorf, BioPhotometer plus, Hamburg, path length 10 mm, material: plastic), the sample was measured against a reaction blank sample containing all reagents. The calibration curve was prepared with *t*BuOOH (in isopropanol/ethanol, 1:1, by vol) in the range of 0.5–20 nmol/ml.

### Analysis of the Oligoprenyl Alcohols and their Oxidation Products by GC and GC-MS

β-Citronellol, nerol, geraniol and *t,t*-farnesol and their oxidation products were monitored on a GC-FID (7890A, Agilent Technologies). An HP-5 capillary column (J & W Scientific Columns, Agilent Technologies), 30 m × 0.32 mm × 0.25 µm, was used. Samples (1 μl) were injected in a split mode. Nitrogen was used as a carrier gas at a flow rate of 1.4 ml/min. The oven temperature was programmed as follows: 100 °C for 3 min, then 8 °C/min to 250 °C and 20 °C/min to 300 °C for 10 min. Inlet and detector temperatures were kept at 250 and 300 °C, respectively. The FID detector was supplied with hydrogen and air at flow rates of 30 ml/min and 400 ml/min, respectively. Calibration curves of oligoprenyl alcohols were used for their quantification as well as their oxidation products. Subsequently, products identified by GC-MS (QP2010S, Shimadzu Corp.) and mass spectra were compared with commercially available products (neral, geranial) or those synthesized with mCPBA standards (6,7-epoxide of citronellol, nerol, geraniol and farnesol; 2,3-epoxide of nerol, geraniol and farnesol; 2,3,6,7-diepoxide of nerol and geraniol; 10,11-epoxide of farnesol), the literature data [19–22] and mass libraries (NIST 147 and Wiley 175). A 30 m × 0.25 mm × 0.25-µm DB-5ms capillary column (Phenomenex) was used with helium as a carrier gas kept at a flow rate of 1.2 ml/min. Samples (1 μl) were injected in a split mode. The oven temperature was programmed as described above. The interface and ion source temperature was set at 250 °C. Mass spectra were collected within the range of *m/z* 40–300 at ionization energy 70 eV.

### Mass Spectrometric EI-MS (70 eV) Data

Citronellal (3,7-dimethyl-6-octenal) *m/z* (%): 41 (100), 69 (62), 55 (40), 95 (33), 67 (22), 84 (18), 121 (17), 111 (14), 81 (14), 154 (0.5); 6,7-epoxycitronellol (6,7-epoxy-3,7-dimethyloctanol) *m/z* (%): 59 (100), 41 (56), 55 (42), 57 (28), 71 (25), 81 (23), 68 (20), 96 (3), 139 (1); 2,3-epoxygeraniol (2,3-epoxy-3,7-dimethyl-6-octenol) *m/z* (%): 41 (100), 67 (48), 69 (45), 55 (28), 109 (27), 61 (21), 82 (18), 81 (14), 95 (12), 139 (5); 6,7-epoxygeraniol (6,7-epoxy-3,7-dimethyl-2-octenol) *m/z* (%): 41 (100), 59 (84), 81 (48), 85 (42), 57 (40), 71 (38), 79 (26), 55 (24), 67 (22), 69 (21), 97 (10), 109 (7); 2,3,6,7-diepoxygeraniol (2,3,6,7-diepoxy-3,7-dimethyloctanol) *m/z* (%): 43 (100), 84 (41), 71 (40), 59 (35), 55 (33), 69 (25), 67 (23), 83 (20), 111 (11), 108 (6); 2,3-epoxynerol (2,3-epoxy-3,7-dimethyl-6-octenol) *m/z* (%): 41 (100), 69 (48), 67 (45), 109 (31), 55 (28), 61 (23), 82 (19), 95 (13), 71 (11); 6,7-epoxynerol (6,7-epoxy-3,7-dimethyl-2-octenol) *m/z* (%): 41 (100), 59 (76), 85 (44), 57 (39), 71 (38), 84 (36), 81 (30), 55 (24), 69 (23), 79 (22), 83 (22), 97 (10), 109 (7); 2,3,6,7-diepoxynerol (2,3,6,7-diepoxy-3,7-dimethyloctanol) *m/z* (%): 43 (100), 71 (42), 84 (33), 59 (33), 55 (30), 67 (22), 69 (17), 111 (8), 108 (4); farnesal (3,7,11-trimethyl-2,6,10-dodecatrienal) *m/z* (%): 69 (100), 41 (69), 84 (40), 81 (16), 67 (14), 55 (14), 93 (8), 95 (6), 109 (5), 121 (5), 136 (5), 220 (0.2); 2,3-epoxyfarnesol (2,3-epoxy-3,7,11-trimethyl-6,10-dodecadienol) *m/z* (%): 69 (100), 41 (92), 81 (43), 67 (24), 55 (23), 109 (16), 93 (14), 79 (12), 95 (10), 107 (10), 121 (6), 135 (2); 6,7-epoxyfarnesol (6,7-epoxy-3,7,11-trimethyl-2,10-dodecadienol) *m/z* (%): 41 (100), 69 (85), 81 (34), 55 (32), 67 (31), 95 (24), 71 (21), 109 (18), 96 (15), 123 (6), 138 (5); 10,11-epoxyfarnesol (10,11-epoxy-3,7,11-trimethyl-2,6-dodecadienol) *m/z* (%): 43 (100), 81 (45), 71 (44), 59 (27), 67 (22), 69 (22), 55 (22), 85 (19), 79 (18), 93 (15), 95 (11), 107 (7), 109 (7), 121 (5), 135 (3).

### Analysis of Solanesol and Pren-9 by GC

Polyisoprenoids were analyzed as trimethylsilyl derivatives on a GC-FID (7890A, Agilent Technologies) [23]. Pyridine and BSTFA/TMCS (99:1) were added to samples dried under a stream of nitrogen; then the mixture was heated for 2 h at 50 °C. Samples were diluted with dichloromethane and injected into the GC apparatus. An HP-5 capillary column (as above) with nitrogen as a carrier gas at a flow rate of 1.6 ml/min was used. The oven temperature was programmed as follows: 220 °C for 2 min, then 25 °C/min to 325 °C for 30 min. Inlet and detector temperatures were kept at 280 and 320 °C, respectively. The FID detector was supplied with hydrogen and air, as described above.

### Analysis of Isoprenoid Alcohols by ESI-MS

The MS spectra of selected samples were recorded on a 4000 Q TRAP mass spectrometer (Applied Biosystems Inc., USA) equipped with an electrospray ion source (TurboIonSpray) and a triple quadrupole/linear ion trap mass analyzer. ESI-MS spectra were obtained in the positive ion mode, in the *m*/*z* range of 100–1,000 or 100–2,000, depending on the investigated compound. The ion source parameters were optimized to obtain the best intensity of the investigated peaks. As a curtain and a nebulizer gas as well nitrogen was employed. The instrument was controlled, and recorded data were processed using the Analyst v. 1.4.2 software package (Applied Biosystems Inc., USA). The samples were dissolved in a mixture of methanol and chloroform (1:1, by vol) and injected into the mass spectrometer by the LC system (Prominence LC-20; Shimadzu).

### Synthesis of Epoxides with mCPBA

Epoxides of short-chain isoprenoids were prepared as described in [24]. Briefly, isoprenoid (10 mmol) dissolved in dichloromethane was stirred with mCPBA (18 mmol) for 24 h at 0 °C. Then the reaction mixture was cooled to −20 °C, filtered and washed with 5 % sodium sulfite and saturated sodium carbonate, then dried with anhydrous sodium sulfate. After removal of the solvent, mono- and diepoxides were isolated from the reaction mixture by column chromatography on silica gel columns eluted with a mixture of hexane and diethyl ether using a step-wise gradient. The identity of products was confirmed by GC/MS analysis as described above.

### Statistical Analysis

The statistical calculations were performed using SPSS Statistics, version 21, IBM Corp., at the *p* < 0.05 level. One-way analysis of variance (ANOVA) in combination with the Duncan test was performed.

## Results

### Degradation of the Substrate

**Fig. 2:**
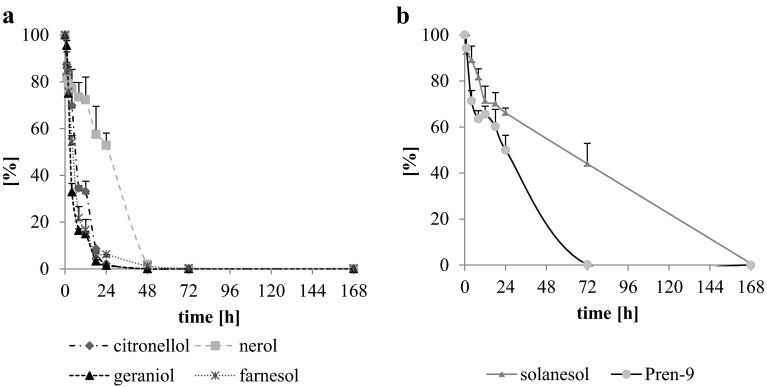
Isoprenoid alcohol degradation rate shown as % of remaining substrate at indicated time points during thermo-oxidation of citronellol, nerol, geraniol, farnesol, solanesol and Pren-9 at 80 °C as determined by GC-FID. Mean values (*n* = 3) + SD are shown

Thermo-oxidation led to decomposition of isoprenoids with simultaneous formation of hydroperoxides (ROOH) followed by decomposition of the latter and formation of secondary oxidation products. Keeping in mind the structure of isoprenoid alcohols subjected to thermo-oxidation, conversion of hydroperoxides toward formation of various products might be expected, including epoxides and aldehydes [14, 20, 25]. Within the first 24 h of thermo-oxidation at 80 °C, the degradation of β-citronellol, geraniol and farnesol was almost complete, leaving only 2.1, 1.5 and 6 % of the initial amount of the unreacted substrate, respectively (Fig. 2a). Among all tested prenols, solanesol was the most stable. After 24 h of oxidation, 66 % of the substrate remained intact, whereas in the case of Pren-9 and nerol, it was 50 and 53 %, respectively (Fig. 2a, b). After 72 h of oxidation of nerol and Pren-9, these substrates were completely oxidized, with 44 % of the initial amount of solanesol still remaining.

### Formation of Oxidation Products

#### Hydroperoxides

**Fig. 3:**
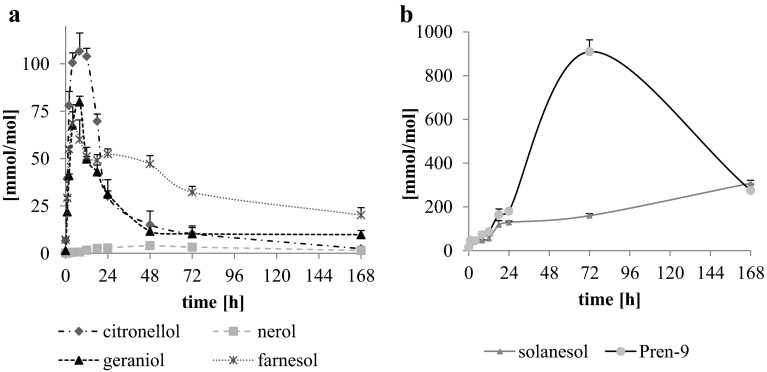
Total content of hydroperoxides (mmol/mol of substrate) formed during thermo-oxidation of citronellol, nerol, geraniol, farnesol, solanesol and Pren-9 at 80 °C as determined by the colorimetric method. Mean values (*n* = 3) + SD are shown

Hydroperoxides were expected as primary oxidation products formed during the thermo-oxidation of isoprenoid alcohols [20, 25]. Indeed, they were detected during oxidation of all analyzed isoprenoid alcohols. The highest content of hydroperoxides was found in the case of medium-chain polyisoprenoids (Fig. 3b), as after 72 h of oxidation of Pren-9 910 mmol of ROOH per mol of substrate was determined, whereas in the case of solanesol after 168 h of thermo-oxidation 308 mmol/mol of ROOH was found. Among short-chain isoprenoids, the highest concentration of hydroperoxides (over 100 mmol/mol of substrate) was reached after 4–12 h of thermo-oxidation of β-citronellol (Fig. 3a). Similarly, for geraniol the maximum amount of ROOH (80 mmol/mol) was reached after 8 h and for farnesol (60–69 mmol/mol) after 4–8 h of thermo-oxidation. The lowest ROOH concentrations were found for nerol, not exceeding 4 mmol/mol at any time point.

#### Epoxides and Aldehydes

**Fig. 4:**
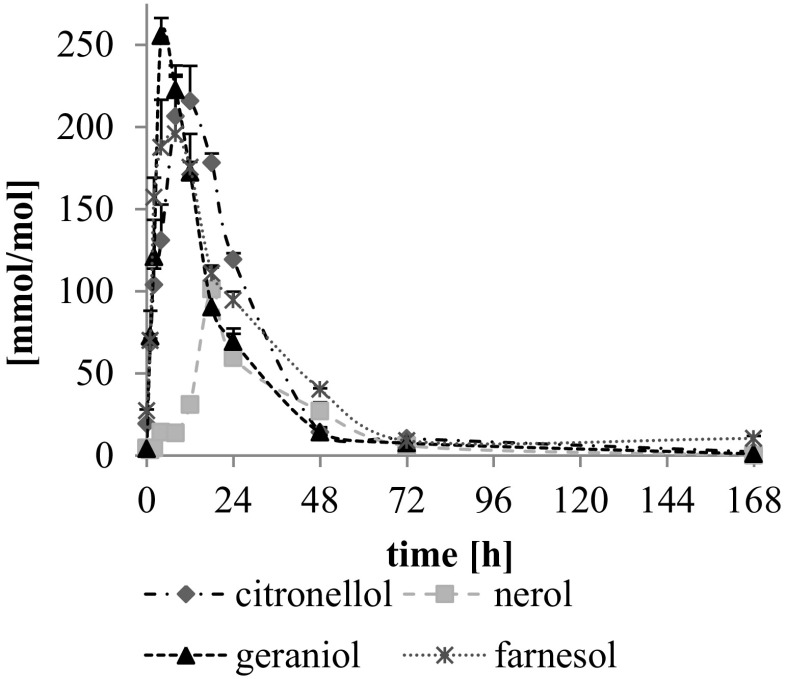
Total content of epoxides and aldehydes (mmol/mol of substrate) formed during thermo-oxidation of citronellol, nerol, geraniol and farnesol at 80 °C analyzed by GC-FID. Mean values (*n* = 3) + SD are shown

**Fig. 5:**
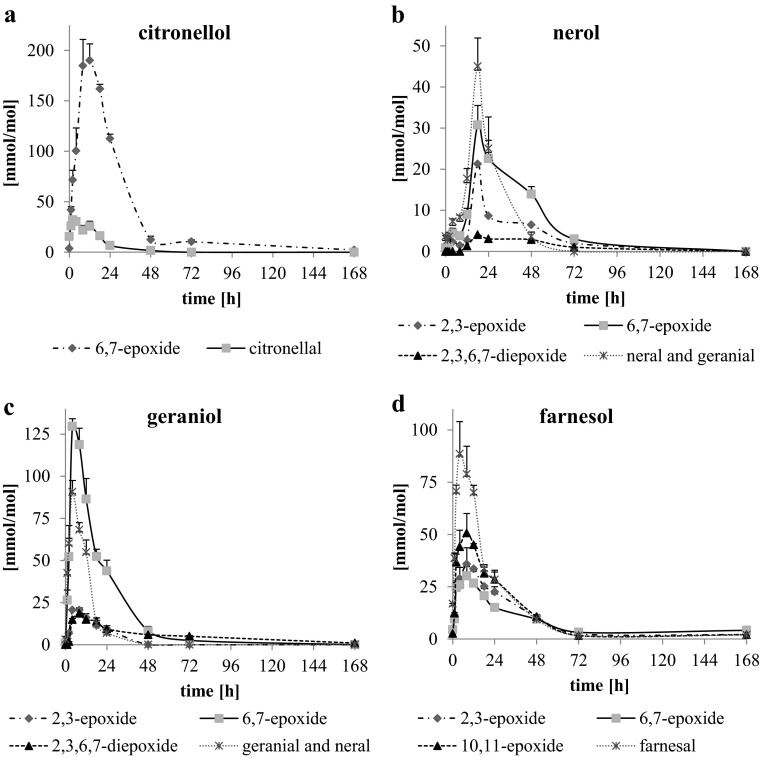
Content of individual oxidation products (mmol/mol of substrate) formed during thermo-oxidation of citronellol, nerol, geraniol and farnesol at 80 °C determined by GC-FID. Mean values (*n* = 3) + SD are shown

Formation of oxidation products of short-chain isoprenoid alcohols, i.e., mono-, diepoxides and aldehydes, during thermo-oxidation was monitored (Figs. 4, 5a–d). The maximum amount of identified oxidation products was observed after 4 h of thermo-oxidation of geraniol (sum of mono-, diepoxides and aldehydes reached 256 mmol/mol of substrate), after 8–12 h in the case of citronellol (sum of monoepoxides and aldehydes in the range of 207–216 mmol/mol) and after 8 h in the case of farnesol (sum of monoepoxides and aldehydes 196 mmol/mol) (Fig. 4). The total amount of identified mono-, diepoxides and aldehydes of nerol was twofold lower than for other short-chain isoprenoids, reaching only 101 mmol/mol after 18 h of thermo-oxidation at 80 °C.

**Fig. 6:**
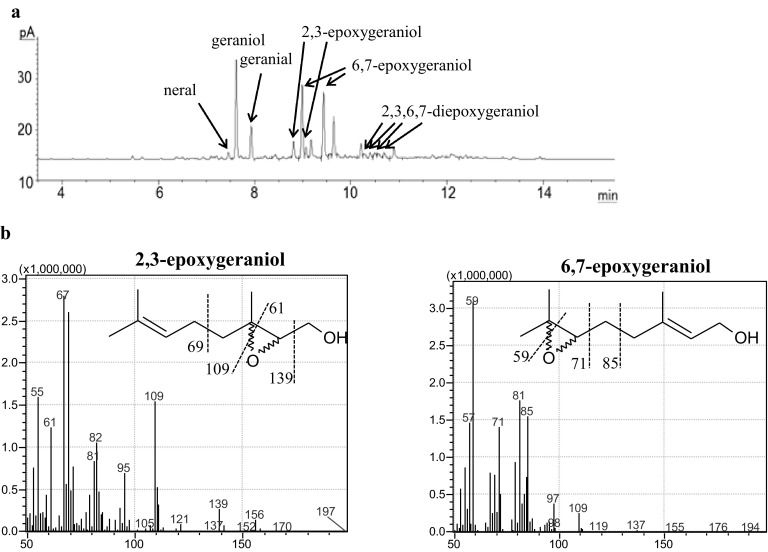
GC-FID chromatogram of geraniol thermo-oxidized for 18 h (**a**). GC-EI/MS mass spectra of 2,3-epoxygeraniol and 6,7-epoxygeraniol (**b**)

Formation of geometric, positional and/or optical isomers of aldehydes and epoxides was observed for the tested short-chain isoprenoids. For example, in the case of geraniol, two positional isomeric monoepoxides were identified, 2,3- and 6,7-epoxides, each in two optical isomers (R) and (S). In Fig. 6a, a GC-FID chromatogram of thermo-oxidized geraniol is presented. GC-EI/MS mass spectra of 2,3-epoxygeraniol and 6,7-epoxygeraniol are presented in Fig. 6b; additionally, the fragmentation pattern is shown. When geraniol and nerol were oxidized, two geometric isomers of aldehyde were identified: geranial (3,7-dimethyl-*trans*-2,6-octadienal) and neral (3,7-dimethyl-*cis*-2,6-octadienal) [20]. The highest amount of total monoepoxides was observed in the case of citronellol, where after 8–12 h of thermo-oxidation 185–190 mmol/mol of 6,7-epoxide was found. The highest amount of aldehydes was found in the case of geraniol (after 4 h 90.7 mmol/mol total amount of geranial and neral) and farnesol (after 4–8 h farnesal in range 79–89 mmol/mol) (Fig. 5a–d). 6,7-Epoxide dominated over 2,3-epoxide of geraniol and nerol, whereas 10,11-epoxide of farnesol was the most abundant among its monoepoxides.

#### Oligomeric Derivatives of Isoprenoids

**Fig. 7:**
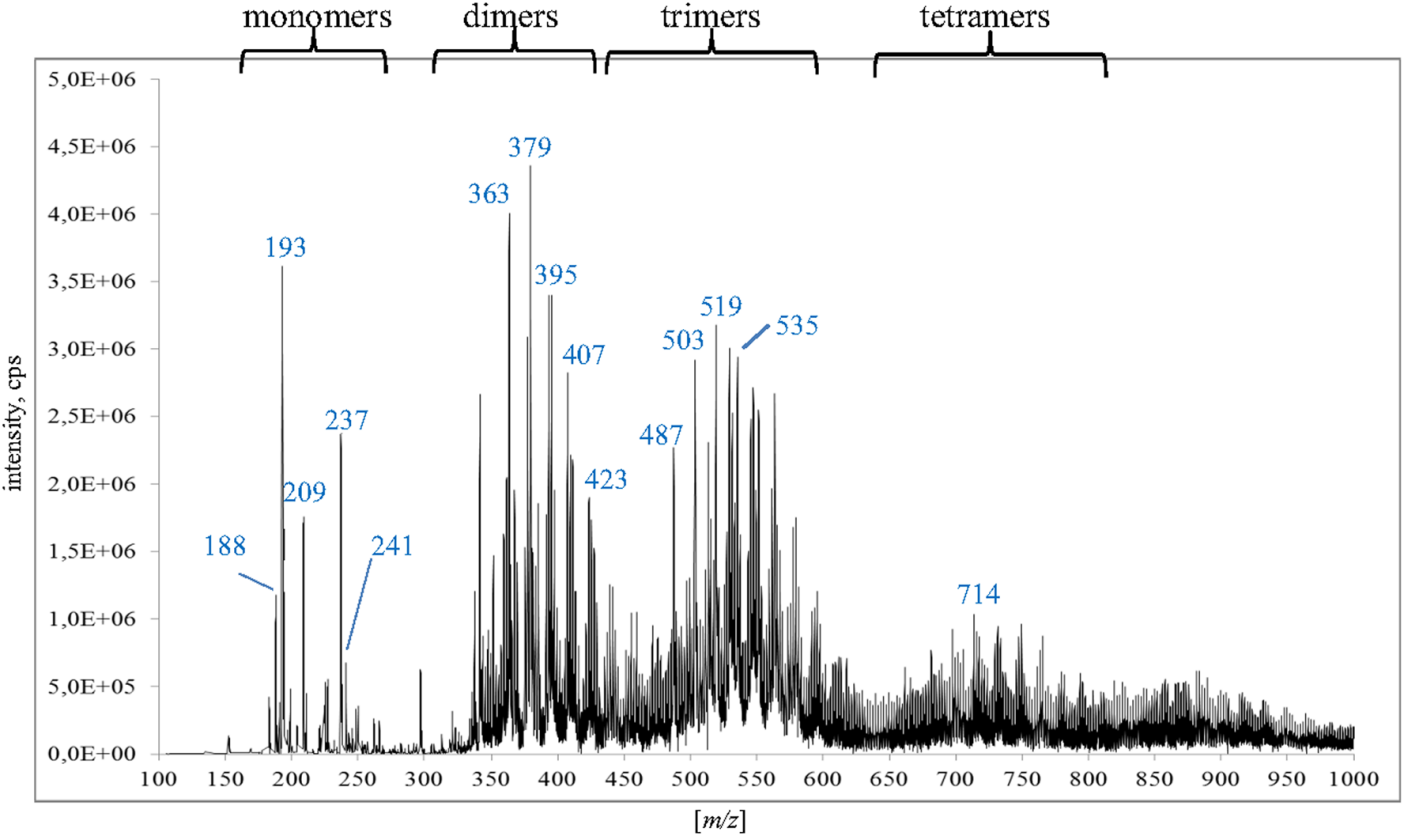
ESI-MS spectrum of thermo-oxidized geraniol. Signals corresponding to oxidized monomers and tentative oligomers are indicated

The possibility of the formation of oligomeric derivatives of isoprenoids upon thermo-oxidation was followed with the aid of the ESI-MS method. Signals corresponding to oxidized monomers were observed; moreover, series of ions corresponding most probably to multiply oxygenated dimers, trimers and tetramers of oligoprenyl alcohols were detected. In Fig. 7, the spectrum of geraniol (C_10_H_18_O) oxidized for 8 h is shown. In the monomeric ion region, the most abundant ions were assigned as sodium and ammonia adducts of oxidized geraniol, e.g., at *m/z* 188, 193, 209, 237 and 241. Moreover, putative dimers, trimers and tetramers were also observed. Similar effects of oligomerization were observed for farnesol, nerol and β-citronellol (data not shown). Furthermore, signals corresponding to the putative dimers were observed for solanesol and Prenol-9 (data not shown).

## Discussion

Profiles of oxidation products were similar for three short-chain isoprenoids: β-citronellol, geraniol and farnesol, reaching the maximum of hydroperoxide and total secondary oxidation product content between 4 and 12 h of thermo-oxidation at 80 °C with simultaneous intense, almost complete degradation of the substrate within the first 24 h of oxidation. Whereas for short-chain isoprenoid in the α-*cis* configuration, i.e., nerol, the maximum content of secondary oxidation products was shifted up to 18 h of thermo-oxidation. Degradation of this substrate was less intense than for short-chain isoprenoids in the α-*trans* configuration, and a very low concentration of hydroperoxides was observed throughout the period of oxidation. Taken together geraniol was more susceptible to oxidation than its *cis* isomer, nerol. Lower reactivity of nerol (α-*cis*) vs. geraniol (α-*trans*) might possibly be explained by the presence of a steric hindrance—an alkenyl group in the molecule of the former compound while a methyl group is present in the *trans* isomer. This larger substituent perturbs the abstraction of the hydrogen atom located at the α-position to the hydroxyl group. According to the literature, this particular hydrogen atom is favorably abstracted upon reaction [20].

However, the opposite relationship was observed for polyprenols, where the rate of degradation was lower for all-*trans* solanesol than for its di-*trans*-poly-cis isomer, Pren-9. A similar phenomenon, higher oxidation stability of *trans* isomers, has been reported for other, previously studied lipids—fatty acids and their esters. Ikeda and Fukuzumi [26] observed that degradation and hydroperoxide formation during autoxidation at 37 °C of methyl oleate (*cis*-18:1) were greater than found for methyl elaidate (*trans*-18:1). They concluded that the different autoxidation rates of these isomers resulted from different affinities of the oxygen molecule to the olefinic double bond of the substrates. Similarly, they observed that the *cis*,*cis* isomer of methyl linoleate (9*cis*,12*cis* 18:2) was oxidized more easily than the 9*trans*, 12*trans* 18:2 isomer [27]. Analogous differences in oxidation rates of *cis* and *trans* geometric isomers were also observed in various testing conditions. Kazuo et al. [28] found that the half-time of decay (50 % of the substrate left) of 9*trans*, 12*trans*-18:2 acid was ca. 36 h, whereas for its *cis* isomer, 9*cis*,12*cis*-18:2, it was ca. 13 h (oxidation initiated by Fe^2+^ in an emulsion at 37 °C). However, they did not observe similar phenomena for monounsaturated oleic (9*cis*-18:1) and elaidic (9*trans*−18:1) acid, nor did Lercker et al. [29] studying oxidation of these monounsaturated acids at 200 °C for 30 min. Moreover, Sargis and Subbaiah [30], studying the oxidation of phosphatidylcholine (PC) liposomes, found that 16:0-18:2 *trans*,*trans*-PC exhibited greater resistance to oxidation than the 16:0-18:2 *cis*,*cis*-PC in respect to degradation of a fluorescently labeled PC and formation of conjugated dienes. These observations were interpreted as higher resistance of *trans* than *cis* unsaturated fatty acids to oxidation reactions due to the lower energy state of a *trans* double bond in comparison to a similarly substituted *cis* double bond as a consequence of the physical distribution of the substituents; *trans* isomers are generally considered less reactive and are usually more stable than *cis* [31]. This is in agreement with data showing that *trans* unsaturated fatty acids are less reactive than their *cis* isomers in substitution and Diels-Alder reactions [30]. Another important consequence of this phenomenon is that intermediates of the lipid oxidation process of *trans* isomers are generally more stable. Teng and Smith [32] found that *trans*,*trans* hydroperoxides of linoleic acid were more stable than their *cis*,*trans* isomers. They also showed that interconversion between positional hydroperoxides of linoleate acid isomers, i.e., rearrangement of the *cis*,*trans* hydroperoxides to their *trans*,*trans* isomers, is to be expected. Both fatty acids and isoprenoid alcohols are susceptible to oxidation, which proceeds through the initial hydrogen abstraction and radical chain reactions and results in the formation of similar products, i.e., hydroperoxides, aldehydes and epoxides. It should be kept in mind, however, that in contrast to unsaturated fatty acids (containing disubstituted double bonds), isoprenoids contain trisubstituted double bonds in their molecules and consequently are thermodynamically more stable (liberate less heat upon hydrogenation) than the UFA.

Despite the influence of the geometry of the double bond on the oxidiative stability of the tested isoprenoids, we have observed that in general oligoprenyl alcohols were more susceptible to thermo-oxidative degradation than polyprenols. These alcohols have different physical properties; however, the temperature of the thermal oxidation (i.e., 80 °C) was set to be above their melting and below their boiling points. Of the tested isoprenoids, solanesol has the highest melting point, i.e., 35 °C, while short-chain isoprenoid alcohols have boiling points above 220 °C, even though as constituents of essential oils they are considered to be relatively volatile [33].

**Fig. 8:**
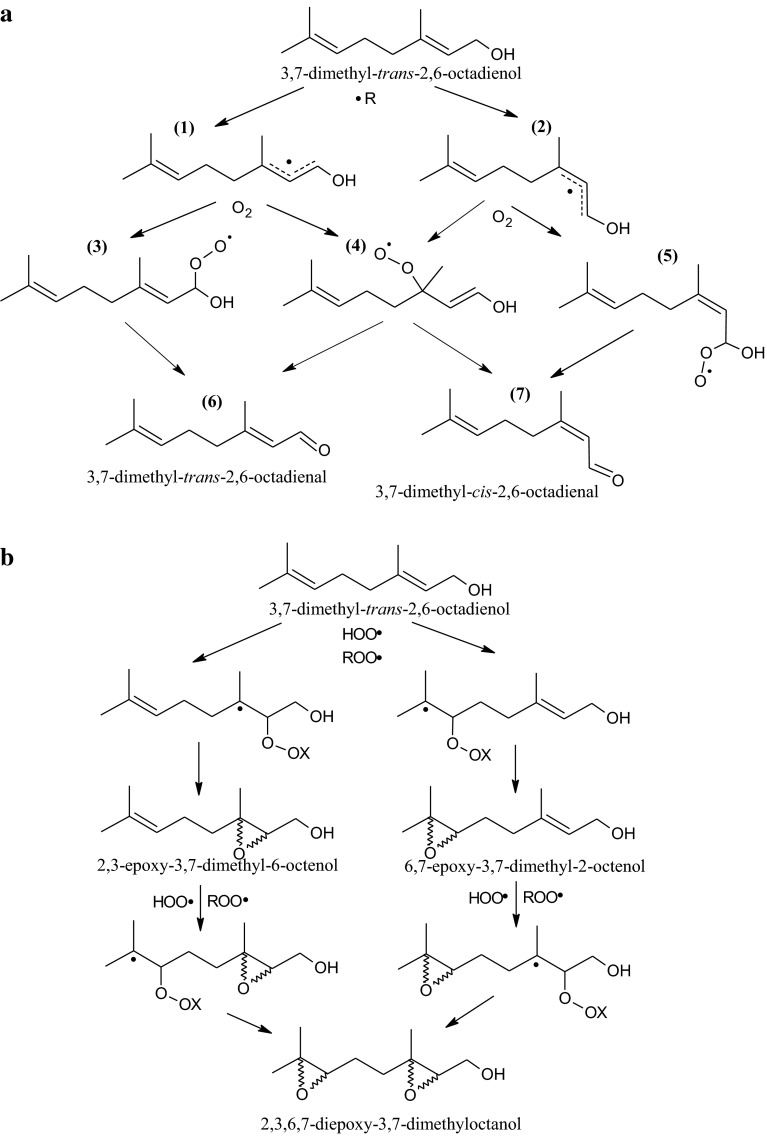
Autoxidation pathways of geraniol, including abstraction of a hydrogen atom (**a**) or by an addition reaction to the double bond (**b**) (according to [14, 20]). X = R or H

Short-chain prenols such as nerol, geraniol or citronellol undergo oxidation initiated by singlet oxygen formed from hydrogen peroxide in the presence of catalytic amounts of manganese (III) porphyrin complexes [34] or molybdate ions [35] as well as by singlet oxygen generated by halogen lamp irradiation in the presence of a photosensitizer (porphyrin) [36] with the formation of hydroperoxides, diols, epoxides, epoxy-diols and epoxy-ketones. These products can also be formed during autoxidation and thermo-oxidation. The mechanism of isoprenoid autoxidation starts from hydrogen abstraction of an allylic hydrogen, followed by the reaction with molecular oxygen O_2_ and radical chain reactions leading to formation of hydroperoxides and subsequently aldehydes and alcohols [14, 20, 25, 37–39]. A scheme of the geraniol autoxidation pathways leading to the formation of the determined compounds is shown in Fig. 8a, b [14, 20]. Similarly to our findings, Hagvall et al. [20] reported the formation of geraniol hydroperoxides along with both isomeric aldehydes, i.e., neral (6 in Fig. 8a) and geranial (7), and epoxygeraniol during autoxidiation of air-exposed geraniol. Although at the initiation step theoretically the formation of five radicals of geraniol is possible by hydrogen abstraction, an experimental study by Hagvall et al. [20] showed that the most easily abstracted allylic hydrogen is the one at Cα to the hydroxyl, which leads to formation of C1–3 radicals (1 and 2). Hagvall et al. [20] and Bäcktorp et al. [14] used computational chemistry to obtain supplemental information on the possible mechanisms of geraniol autoxidation, demonstrating that the formation of other geraniol radicals is less feasible. For instance, the enthalpy change (ΔΔH) for forming the C5–7 radical is higher by 6 kcal/mol in relation to the calculated value for forming the C1–3 radical. Moreover, the C5–7 radical is less stable than the C1–3 radical, but some of the products derived from the C5–7 radical were still observed by Hagvall et al. [20], such as 7-hydroperoxide or 1,7-diol of geraniol. The three other radicals, two C2–4 radicals and a C7 radical, are higher in energy (ΔΔH in the range of 8–11 kcal/mol in relation to the C1–3 radical), and no oxidation products derived from these radicals were observed by Hagvall et al. [20]. Hydrogen abstraction can be facilitated by numerous radicals such as R·, RO·, ROO·, but also HOO· or ·OH [14, 39]. Next to the hydrogen abstraction, an important autoxidation reaction is an addition reaction to the double bond of such species as HOO· and ROO·, leading to epoxidation of the isoprenoid molecule (Fig. 8b) with release of the OH· or RO· radical. Our results showed that the double bond most distant to the OH group was preferentially epoxidized, since 6,7-epoxides were the most abundant in the case of both nerol and geraniol and 10,11-epoxide in the case of farnesol (Fig. 5b–d). Although formation of geometrically isomeric aldehydes as a product of geraniol oxidation has been previously observed [14, 20] (Fig. 8a), we found different ratios of aldehydes in the *trans*/*cis* configuration depending on the stereochemistry of the starting compound. Geranial (6) was more abundant than neral (7) by ca. fourfold during geraniol oxidation, whereas neral was dominant only by ca. twofold over geranial during nerol oxidation (data not shown). This can be explained by free rotation of the single bond adjacent to the double bond in the C1–3 radical (1 and 2) or in the C1 3-hydroperoxy radical (4) [20] of geraniol and nerol. Because the *cis* configuration is thermodynamically less favored than the *trans* configuration, single bond rotation will be expected to occur more frequently during nerol than geraniol oxidation.

The amount of oxidation products of isoprenoids determined in each of time points is a result of their simultaneous formation and degradation. As presented in Fig. 8, less stable hydroperoxides (and hydroperoxide radicals) are transformed to other more stable oxidation products such as aldehydes, epoxides and diols [14, 37]. However, similarly to what is observed during the thermo-oxidation of other important lipids, e.g., fatty acids, triacylglycerol or sterols, further transformations should occur [40–45]. Primary and secondary oxidation products of lipids are degraded to low-molecular-weight products, e.g., olefins, alcohols, ketones, aldehydes, acids, etc. These volatile products are formed by cleavage of the epoxide ring, by β-scission of an adjacent C–C bond to the hydroperoxide group or alkoxy group and other rearrangements [40, 41]. On the other hand, if oxidation of a lipid occurs at high temperature, high-molecular-weight products such as oligomers and polymers are formed [42, 44–46]. Therefore, the observed degradation of the analyzed oxidation products (i.e., hydroperoxides, epoxides and aldehydes) of the tested isoprenoids during thermo-oxidation could be explained by their transformations to other products with lower and higher molecular weights corresponding to fragmentation and oligomerization of the substrates, respectively. This hypothesis was confirmed by direct ESI-MS analysis, as next to ions corresponding to sodium adducts of oxidized monomers, products at higher *m/z* values—tentatively considered as oligomers with multiple sites of oxygenation—were also observed (Fig. 7). Oligomers, multiply oxidized, were found previously as typical derivatives observed for thermo-oxidized lipids, such as triacylglycerols, fatty acids or sterols [45–47].

Higher amounts of hydroperoxides determined by the colorimetric ferrous thiocyanate method in thermo-oxidized solanesol and Pren-9 samples than in the case of short-chain isoprenoids can be explained by the formation of derivatives with several hydroperoxy groups. Compounds built from two or three isoprene units are expected to form primarily monohydroperoxides, quickly transformed to more stable derivatives; however, longer isoprenoid chains provide an increased number of possible sites of hydroperoxidation. Nakagawa et al. [25], after oxidation of squalene, found mainly monohydroperoxides along with di- and trihydroperoxides, whereas Mountfort et al. [48] identified up to pentahydroperoxides of squalene. Each hydroperoxy group will react with Fe^2+^, leading to overestimation of the hydroproxide content determined by the colorimetric method. Another factor further affecting spectrophotometric readings are reactive oxygen species (reacting with Fe^2+^), such as hydrogen peroxide and its precursor, the hydroperoxyl radical, which can be formed when hydroperoxides transform to aldehydes (Fig. 8a) [20]. Despite these drawbacks, the colorimetric ferrous thiocyanate method is especially useful when the results are compared between compounds with the same number of double bonds, i.e., geraniol vs. nerol or solanesol vs. Pren-9.

Summarizing, thermo-oxidation led to decomposition of the tested short-chain isoprenoids and polyprenols with simultaneous formation of oxidized derivatives, such as hydroperoxides, monoepoxides, diepoxides and aldehydes. Moreover, as the result of thermo-oxidation, fragmented molecules of Pren-9 and solanesol as well as possible oligomers of all tested isoprenoids were formed. Profiles of the oxidation products were similar for three short-chain isoprenoids: β-citronellol, geraniol and farnesol, with simultaneous intense, almost complete degradation of the substrate within the first 24 h of oxidation. Unexpectedly, nerol, a short-chain isoprenoid in the *cis* configuration, was more oxidatively stable than its *trans* isomer, geraniol. The relative susceptibility of polyprenols to thermo-oxidation followed the roles generally observed for *cis* and *trans* isomers since mainly-*cis* prenol-9 was more susceptible to thermo-oxidation than its all-*trans* isomer, solanesol. The present study shows that compounds differing in the number of isoprene units and the geometry of double bonds have different susceptibilities to thermo-oxidation.

Taken together, these results experimentally confirm that both short- and long-chain polyisoprenoid alcohols are prone to chemical oxidation. It can be speculated that oxidized isoprenoids might be formed in the cells because of chemical processes driven by oxidative agents (e.g., reactive oxygen species). Such derivatives might contribute to the regulation of metabolic processes. Moreover, after optimization of the reaction conditions, controlled thermo-oxidation might be considered an efficient method for the synthesis of biotechnologically important isoprenoid derivatives.
